# High-expressed ACAT2 predicted the poor prognosis of platinum-resistant epithelial ovarian cancer

**DOI:** 10.1186/s13000-023-01435-4

**Published:** 2024-01-04

**Authors:** Jinfeng Wang, Zhe Yang, Han Bai, Lanbo Zhao, Jing Ji, Yadi Bin, Yu Liu, Siyi Zhang, Huilian Hou, Qiling Li

**Affiliations:** 1 Department of Obstetrics and Gynecology, First Affiliated Hospital of Xi’an Jiaotong University, 277 Yanta West Road, Xi’an, Shaanxi 710061 China; 2 Department of Pathology, First Affiliated Hospital of Xi’an Jiaotong University, 277 Yanta West Road, Xi’an, Shaanxi 710061 China; 3 The MED-X Institute, The First Affiliated Hospital of Xi’an Jiaotong University, Western China Science and Technology Innovation Harbor, Building 21, Xi’an, 710000 China

**Keywords:** ACAT2, Chemo-response, Prognosis, Epithelial ovarian cancer

## Abstract

**Background:**

Acetyl-CoA acetyltransferase 2 (ACAT2) is a lipid metabolism enzyme and rarely was researched in epithelial ovarian cancer (EOC).

**Methods:**

ACAT2 expressions were confirmed in two pairs of cell lines (A2780 and A2780/DDP, OVCAR8 and OVCAR8/DDP) from Gene Expression Omnibus database by bioinformatics analysis, and in A2780 and A2780/DDP cell lines by quantitative real-time polymerase chain reaction and western blotting. Tissue samples were stained by immunohistochemistry and scored for ACAT2 expression. The relationships between ACAT2 expression and clinicopathological characteristics were analyzed by χ^2^ test. The prognosis of ACAT2 was analyzed by the log-rank tests and Cox regression models.

**Results:**

ACAT2 was remarkably upregulated in the above drug-resistant cell lines by mRNA (all *P* < 0.05) and protein expression (*P* = 0.026) than those in sensitive ones. Patients were classified as ACAT2-high (n = 51) and ACAT2-low (n = 26) according to immunohistochemical score. ACAT2 expression had a significantly inverse correlation with FIGO stage (*P* = 0.030) and chemo-response (*P* = 0.041). A marginal statistical significance existed in ACAT2 expression and ascites volume (*P* = 0.092). Univariate analysis suggested that high-expressed ACAT2 was associated with decreased platinum-free interval (PFI) (8.57 vs. 14.13 months, *P* = 0.044), progression-free survival (PFS) (14.12 vs. 19.79 months, *P* = 0.039) and overall survival (OS) (36.89 vs. 52.40 months, *P* = 0.044). Multivariate analysis demonstrated that ACAT2 expression (hazard ratio = 2.18, 95% confidence interval: 1.15–4.11, *P* = 0.017) affected OS independently, rather than PFI and PFS.

**Conclusion:**

The expression of ACAT2 in A2780/DDP and OVCAR8/DDP was higher than the corresponding A2780 and OVCAR8. High-expressed ACAT2 was associated with advanced FIGO stage, chemo-resistance, and decreased PFI, PFS and OS. It was an independent prognostic factor of OS in EOC.

**Supplementary Information:**

The online version contains supplementary material available at 10.1186/s13000-023-01435-4.

## Introduction

Ovarian cancer (OC) is the most lethal gynecologic malignancy, with a 5-year survival rate of 39–47% [1, 2]. Its poor survival rate comes from unclear pathogenesis, delayed diagnosis and primary or progressive chemo-resistance [3]. Platinum-based chemotherapy is the backbone of OC treatment, and benefiting 70% of patients [4]. While 70–85% among them will relapse within 3 years [5] and develop chemo-resistance over time leading to death [6]. Some researchers have reported that immunotherapy [7] and adoptive cell therapy [8] are able to reverse platinum-resistance. However, their response rates in OC are low, ranging from 8–15% [9, 10]. Similar results are observed in combination with immunotherapy [11] and in the application of bevacizumab [12] and poly (ADP-ribose) polymerase inhibitors for first-line maintenance treatment. This may be attributed to the heterogeneity of ovarian cancer and lack of the most representative marker to predict chemo-response [13], which makes clinical treatment challenging.

Recently, some researchers have constructed chemo-response models of epithelial ovarian cancer (EOC) from different perspectives, such as sequencing data from tissue [1, 14, 15] and fecal [16], clinical data including image information [17, 18], and the combination of both data mentioned above [19, 20]. All the models were characterized by small sample sizes and limited false positive rates. There is still an unmet need of new biomarkers to predict individual disease course and chemoresistance. Platinum-resistance contains complex mechanisms involving multiple genes, steps and pathways due to high genomic instability [15]. Studies have reported that microenvironment [21], epigenetics [22], cancer stem cells [23] and related markers are involved in platinum-resistance of OC. So far, no reliable marker has been found to predict chemo-resistance. Thus, it is crucial to identify the most dominant resistance mechanisms by a such biomarker before treatment.

Our previous study constructed a chemo-response model involving three genes: acetyl-CoA acetyltransferase 2 (ACAT2), anterior gradient 2 (AGR2) and heat shock-related 70-kDa protein 2 (HSPA2) [1]. Downregulation of AGR2 was reported to relate to chemo-resistance of EOC [24], which was consistent with ours [1]. Rare studies of HSPA2 and ACAT2 were reported in EOC and chemo-resistance.

ACAT2 is a subtype of Acetyl-coenzyme A acetyltransferase (ACAT), which is a membrane-bound enzyme and plays important roles in lipid metabolism [25, 26]. Recent years, many studies have found that aberrantly expressed ACAT2 is associated with carcinogenesis and progression, such as hepatocarcinoma [26], clear cell renal cell carcinoma [27], colorectal cancer [28], breast cancer [29], and so on. ACAT1 is another subtype of ACAT [26]. Ayyagari’s study suggested that ACAT1 inhibition had anti-tumor effects and was associated with cisplatin (DDP) sensitivity in EOC [30]. However, rare researches have been done between ACAT2 expression and drug-resistance in EOC. So, ACAT2 was used as the target for this study.

Our previous study suggested that ACAT2 was upregulated in platinum-resistant EOC and related to shorter survival [1]. But it lacked validation both in vitro and vivo. In this study, we utilized EOC patients’ tissue, a pair of ovarian endometroid adenocarcinoma cell lines (A2780 and A2780/DDP) and Gene Expression Omnibus (GEO) database to confirm the above conclusions. We analyzed the correlations between ACAT2 expression and various clinicopathological characteristics related to prognosis.

## Materials and methods

### Tissue samples

From January 2016 to December 2020, 77 patients with primary EOC treated at the First Affiliated Hospital of Xi’an Jiaotong University were enrolled. There were 72 serous carcinomas with 62 high-grade and 10 low-grade, 2 high-grade serous with endometrioid cancer, 2 high-grade serous with mucinous carcinoma and 1 high-grade clear cell carcinoma. All patients underwent tumor cytoreductive surgery and platinum-based chemotherapy after surgery. Clinicopathological and follow-up data were completed; Patients were excluded with other tumor histories or preoperative radio-chemotherapy. Tissues were formalin-fixed and paraffin-embedded for histopathologic diagnosis and immunohistochemical study. The ethics committee of the First Affiliated Hospital of Xi’an Jiaotong University approved this study (No. XJTU1AF2022LSK-182). Informed consent was obtained from each patient.

### Cell lines

The human OC cell lines A2780 and A2780/DDP were donated by Prof. Le Zhao of Center for Translational Medicine, the First Affiliated hospital of Xi’an Jiaotong University (Xi’an, Shannxi). A2780 and A2780/DDP cell lines were cultured in RPMI 1640 medium (Gibco), containing 10% fetal bovine serum (BI) and 1% penicillin-streptomycin (Cytiva) in a humidified incubator at 37 °C with 5% CO_2_. The medium of A2780/DDP contained 1ug/mL cisplatin (Selleck) to maintain tolerance.

### ACAT2 microarray data information

Microarray data information of cisplatin-sensitivity and cisplatin-resistance OC cell lines were obtained from NCBI-GEO (https://www.ncbi.nlm.nih.gov/geo/) database. When “cisplatin-resistance” was used as a keyword to perform query, we selected the original ovarian cancer studies of RNA assay in *Homo sapiens* for analysis. The expression microarray datasets GSE45553, GSE15709 and GSE33482 were downloaded.

### Cell viability

Cells (5 × 10^4^/mL) were seeded into 96 well plates with 100uL per well. After 24 h, different concentrations (0, 0.25, 0.5, 1, 2, 4, 8, 16, 32 ug/mL) of cisplatin were added and incubated for 48 h. Cell viability was measured by cell counting kit-8 (CCK8, Targetmol). A complete medium containing 10% CCK8 was added into each well of plate, which was placed in a dark environment at 37℃ for 2–4 h. Then, a microplate reader (KHB ST-360, shanghai) was used to detect the absorbance (OD value) at 450 nm. Cells incubated with 10% CCK8 complete medium were set as control. The wells only containing 10% CCK8 complete medium were used as blank. Cell viability = (OD _experiment_ - OD _blank_) / (OD _control_ - OD _blank_).

### ACAT2 expression detected by qRT-PCR

Total RNA was extracted from A2780 and A2780/DDP cell lines using TRIzol reagent (TIANGEN, China) and treated with RNase-free. Reverse transcription was conducted to obtain cDNA from 1ug RNA utilizing cDNA Synthesis Kit (Novozan) and propagated using ChamQ Universal SYBR qPCR Master Mix (Novozan) in real-time PCR (QuantStudio Dx, Life Technologies). The primers were synthesized by Tsingke Biotechnology Co., Ltd. (China). The sequences of primers used are as follows: 5’-GCCTTCCATTATGGGAATAGGA-3’ and 5’-GACCTTCTCTGGGTTTAATCCA-3’ for ACAT2; 5’-GGAGTCCACTGGCGTCTTCA-3’ and 5’-GTCATGAGTCCTTCCACGATACC-3’ for GAPDH. The comparative threshold cycle (2^−ΔΔCt^) equation was applied to calculate the relative ACAT2 mRNA expression as well as compare the expression of GAPDH as a loading control.

### ACAT2 expression detected by western blotting

Western blotting was performed in the above two cell lines. Cells were lysed on ice for 30 min by RIPA lysis buffer containing 1 μm phenylmethanesulfonyl fluoride (Solarbio). The lysates were centrifuged at 13,000 rpm for 30 min to obtain total protein. Its concentration was quantified by a bicinchoninic acid assay (Solarbio). Protein samples were separated by 10% sodium dodecyl sulfate–polyacrylamide gel and transferred to polyvinylidene fluoride membrane (0.45 μm, Merck Millipore). After being blocked with 5% defatted milk powder (BD) for 1 h at room temperature, the primary antibodies (anti-rabbit ACAT2, 1:5000, ab131215, Abcam; anti-mouse GAPDH, 1:50000, 60004-1-Ig, Proteintech) were incubated with membrane overnight at 4℃. After being washed for 3 times, membranes were incubated with horseradish peroxidase-conjugated secondary antibodies (anti-mouse, 1:5000, SA0001-1, Proteintech; anti-rabbit, 1:3000, GB23303, Servicebio) for 1 h at room temperature and visualized with a high ECL detection reagent (AR) using ImageQuant 800 (Amersham).

### Immunohistochemistry (IHC)

Paraffin-embedded tissues were serially sectioned at 4 μm thickness. They were baked at 65℃ for 2 h, deparaffinized in xylene, and rehydrated through graded alcohol. Subsequently, slices were placed in 3% hydrogen peroxide, heated in 1x sodium citrate antigen retrieval buffer (pH 6.0) by pressure cooker and blocked with 5% bovine serum albumin (Solarbio). The slices were incubated with rabbit anti-ACAT2 monoclonal antibody overnight at 4℃ (1:300; ab131215, Abcam) and then with the MaxVision-HRP rabbit/mouse antibody (Maixin) at room temperature for 1 h. Diaminobenzidine (Servicebio) was used as the final chromogen. Hematoxylin (Servicebio) was applied to counterstain.

All slices were evaluated by two experienced pathologists in a blinded manner. For the assessment of ACAT2, five high-power fields in each specimen were selected randomly, and cytoplasm staining was examined. Immune score equaled to the percentage of positive cells (0, ≤ 5%; 1, 6 − 25%; 2, 26 − 50%; 3, 51 − 75%; 4, 76 − 100%) multiplied by the staining intensity (0, negative; 1, weak; 2, moderate; 3, strong). Immune scores of 0–4 are defined as low expression and 5–12 as high expression [31].

### Outcome measurement

Platinum-free interval (PFI) was measured from the date of last platinum-based chemotherapy to that of disease progression. PFI > 6 months is defined as platinum-sensitivity and < 6 months as platinum-resistance. Survival information was acquired by telephone and medical records. PFS is defined as the time of surgery to the first disease progression or death due to any causes or the date of last follow-up. Overall survival (OS) is defined as the time from surgery to death due to any causes or the date of last follow-up. The follow-up ended on March 14, 2023. Survival time was calculated in months.

### Statistical analysis

Categorical variables were presented as frequencies. Continuous variables were expressed as means ± standard deviation. The associations between ACAT2 and clinicopathological parameters were evaluated by χ^2^ test. Prognostic factors for PFI, PFS and OS were performed by the log-rank test for univariate analysis and Cox-proportional hazards regression model for multivariate analysis. Survival curves were plotted using Kaplan-Meier method. Differences between curves were analyzed by the log-rank test. All experiments were repeated for three times. Paired data was compared by t-test. *P <* 0.05 was considered as statistically significant. Statistical analyses were performed using SPSS version 22.0 (IBM Corp., Armonk, NY, USA), GraphPad Primer 8.0 and R 3.6.1.

## Results

### The expression of ACAT2 in ovarian cancer cell lines

**Fig. 1:**
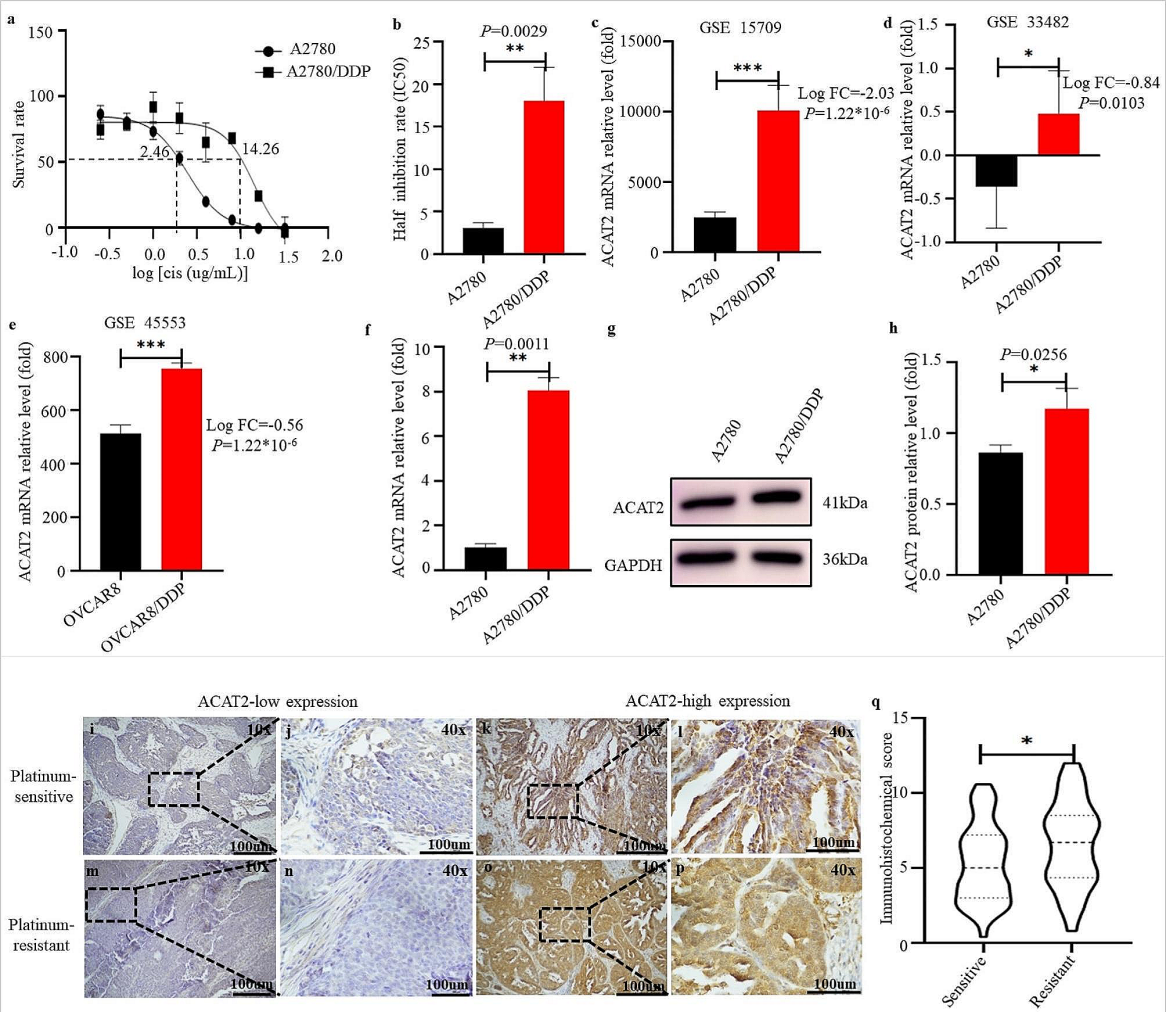
ACAT2 expression level was detected by bioinformatics analysis, qRT-PCR and western blotting in ovarian cancer cell lines and stained by immunohistochemistry with anti-ACAT2 antibody in ovarian cancer tissues. (**a**) The cisplatin dose-response curve in A2780 and A2780/DDP cells. They were exposed to DDP with different concentrations (0, 0.25, 0.5, 1.0, 2.0, 4.0, 8.0, 16.0, 32.0 ug/mL) for 48 h. Cell viability was measured by CCK8. (**b**) Semi-quantitative analyses of the half inhibition rate of A2780 and A2780/DDP. (**c-e**) The relative ACAT2 mRNA expression level in A2780 and A2780/DDP from GSE 15709 and GSE 33482 (data normalization processing), and in OVCAR8 and OVCAR8/DDP from GSE 45553. (**f**) The relative expression level of ACAT2 mRNA in A2780 and A2780/DDP. It was indicated as a normalization of GAPDH in each sample to the control. (**g**) The western blotting of ACAT2 in A2780 and A2780/DDP cells. GAPDH was used as the endogenous reference. (**h**) The relative expression level of ACAT2 protein was indicated as a normalization of GAPDH in each sample to the control. (**i**) ACAT2-low expression in platinum-sensitive (x100); (**j**) ACAT2-low expression in platinum-sensitive (x400); (**k**) ACAT2-high expression in platinum-sensitive (x100); (**l**) ACAT2-high expression in platinum-sensitive (x400); (**m**) ACAT2-low expression in platinum-resistant (x100); (**n**) ACAT2-low expression in platinum-resistant (x400); (**o**) ACAT2-high expression in platinum-resistant (x100); (**p**) ACAT2-high expression in platinum-resistant (x400). (**q**) Immunohistochemical semi-quantitative scores of ACAT2 expression in OC tissues

A2780 and A2780/DDP were treated with different concentrations of cisplatin for 48 h and the values of half maximal inhibitory concentration (IC50) were measured by CCK8. The IC50 of A2780 and A2780/DDP were 2.46 and 14.26 ug/mL (Fig. 1a); The semi-quantitative analyses of half inhibition rate in A2780 and A2780/DDP cell lines were shown in Fig. 1b. The expression of ACAT2 confirmed in A2780/DDP and OVCAR8/DDP was significantly higher at mRNA level by bioinformatics analysis (*P* < 0.05, Fig. 1c–e) and qRT-PCR (*P* < 0.01, Fig. 1f) than the corresponding A2780 and OVCAR8. The protein level of A2780/DDP was higher than A2780 (*P* = 0.026, Fig. 1g-h).

### ACAT2 expression in relation to patients’ clinicopathological characteristics

ACAT2 is mainly expressed in the cytoplasm and occasionally in the nucleus. ACAT2 expression was shown in Fig. 1i-p. The IHC score of 4 is used as a cutoff value for ACAT2-low (n = 26) and ACAT2-high (n = 51) expression. The IHC semi-quantitative scores of ACAT2 expression in resistant OC tissues were higher than that in sensitive those (6.58 ± 2.88vs 5.31 ± 2.59, *P* = 0.049), which were shown in Fig. 1q. The correlations between ACAT2 expression and 77 EOC patients’ clinicopathological characteristics were presented in Table 1. Results showed that ACAT2 expression was significantly associated with International Federation of Gynecology and Obstetrics (FIGO) stage (*P* = 0.030) and chemo-response (*P* = 0.041). There was marginal statistical significance between ACAT2 expression and ascites volume (*P* = 0.092), but no relationship to other clinicopathological characteristics (all *P* > 0.05).

**Table 1:** Correlations between ACAT2 expression and the clinicopathological characteristics

		ACAT2 expression			
	Total (n = 77)	Low (n = 26)	High (n = 51)	χ^2^ value	*P*
**Mean age at diagnosis (years)**				1.08	0.298
< 55	41	16	25		
≥ 55	36	10	26		
**FIGO stage**				7.01	0.030
I	3	3	0		
II	2	0	2		
III - IV	72	23	49		
**Weight (kg)**				0.06	0.812
< 55	40	14	26		
≥ 55	37	12	25		
**Height (cm)**				2.32	0.127
< 158	36	9	27		
≥ 158	41	17	24		
**Pathological type**				0.99	0.610
Serous carcinoma	72	24	48	0.232	0.630
High-grade	62	20	42		
Low-grade	10	4	6		
Mix serous carcinoma	4	2	2		
Clear cell carcinoma	1	0	1		
**Grades**				0.20	0.655
High	67	22	45		
Low	10	4	6		
**Tumor size(cm)**				＜0.01	0.949
< 8.5	47	16	31		
≥ 8.5	30	10	20		
**Chemo-response**				4.17	0.041
Sensitivity	47	20	27		
Resistance	30	6	24		
**CA125 (U/ml)**				＜0.01	0.942
< 2475	59	19	40		
≥ 2475	16	5	11		
**HE4(pmol/L)**				2.56	0.278
< 733	40	11	29		
≥ 733	29	13	16		
Miss	8	2	6		
**Ascites volume (ml)**				2.84	0.092
< 1492	37	9	28		
≥ 1492	40	17	23		
**Surgical satisfaction**				3.98	0.136
R0	12	7	5		
R1	50	14	36		
R2	15	5	10		

*Note*: ACAT2: Acetyl-CoA acetyltransferase 2; FIGO: International Federation of Gynecology and Obstetrics; CA125: carbohydrate antigen 125; HE4: human epididymal protein 4

### Univariate analysis of prognosis for PFI, PFS and OS

Table 2 showed the comparisons between survival outcomes and various clinicopathologic parameters by Kaplan-Meier method.

**Table 2:** Prognostic factors for platinum-free interval (PFI), progression-free survival (PFS) and overall survival (OS) selected by kaplan-meier analysis

	PFI				PFS				OS			
	Mean (months)	95% CI	χ2	*P*	Mean (months)	95% CI	χ2	*P*	Mean (months)	95% CI	χ2	*P*
Mean age at diagnosis (years)												
< 55	11.57	8.18–14.96	1.25	0.263	17.19	13.89–20.50	1.17	0.280	47.22	38.09–56.36	1.56	0.211
≥ 55	9.18	5.96–12.40			14.71	11.50-17.92			37.12	29.61–44.63		
**FIGO stage**												
I-II	19.13	11.45–26.82	8.79	0.012	24.03	16.83–31.24	13.08	0.001	56.61	42.78–70.43	5.08	0.079
III	10.37	7.84–12.90			16.11	13.63–18.59			43.42	36.40-50.44		
IV	2.85	0.00-6.42			7.03	2.76–11.29			24.76	10.36–39.17		
**Weight (kg)**												
< 55	12.08	8.71–15.44	1.63	0.202	17.81	14.58–21.04	1.74	0.187	45.97	37.24–54.71	0.49	0.486
≥ 55	8.69	5.48–11.91			14.11	10.86–17.36			38.2	30.38–46.02		
**Height (cm)**												
< 158	11.01	8.42–13.60	0.11	0.744	16.19	13.70-18.68	0.00	0.986	43.9	34.51–53.29	0.09	0.761
≥ 158	9.96	6.15–13.77			15.90	12.11–19.68			42.73	33.84–51.61		
**Pathological type**												
Serous carcinoma	10.86	8.38–13.33	4.08	0.130	16.45	14.02–18.89	0.04	6.300	45.00	38.31–51.68	10.99	0.004
Mix serous carcinoma	3.78	0.00-8.18			9.03	5.16–12.90			20.25	9.68–30.82		
Clear cell carcinoma	7.83	7.83–7.83			13.73	13.73–13.73			17.00	17.00–17.00		
**Grades**												
High	9.77	7.49–12.05	2.09	0.148	15.38	13.15–17.62	2.17	0.141	41.85	34.89–48.81	1.16	0.281
Low	15.01	5.30-24.72			20.39	10.71–30.06			55.59	36.96–74.21		
**Tumor size (cm)**												
< 8.5	10.83	7.48–14.19	0.39	0.531	16.33	13.02–19.64	0.19	0.664	43.78	34.89–52.66	0.02	0.887
≥ 8.5	9.85	6.83–12.87			15.57	12.60-18.53			42.99	33.92–52.06		
**Chemo-response**												
Sensitivity	15.95	13.09–18.81	102.72	< 0.001	21.19	18.41–23.97	65.09	< 0.001	51.08	43.34–58.82	12.60	< 0.001
Resistance	1.83	0.98–2.69			7.95	6.31–9.59			26.65	20.61–32.68		
**CA125 (U/ml)**												
< 2475	11.24	8.69–13.79	1.67	0.197	16.65	14.13–19.17	1.29	0.255	42.60	35.51–49.69	0.18	0.676
≥ 2475	7.45	1.75–13.16			13.68	8.05–19.31			40.47	30.29–50.66		
**HE4 (pmol/L)**												
< 733	11.66	7.95–15.36	1.73	0.422	17.32	13.71–20.93	1.62	0.446	50.94	40.55–61.32	7.67	0.022
≥ 733	9.42	6.17–12.68			14.89	11.64–18.13			38.95	31.43–46.48		
Miss	8.14	2.46–13.82			13.75	7.86–19.64			25.03	11.00-39.07		
**Ascites volume (ml)**												
< 1492	12.13	8.79–15.46	3.92	0.048	17.51	14.20-20.82	3.19	0.074	49.41	40.32–58.50	6.14	0.013
≥ 1492	8.09	5.05–11.13			13.95	10.98–16.92			32.63	26.88–38.39		
**Surgical satisfaction**												
R0	22.67	16.30-29.04	12.73	0.002	26.79	20.74–32.83	10.68	0.005	64.98	52.18–77.79	7.36	0.025
R1	7.7	5.70–9.71			13.54	11.42–15.66			37.23	30.14–44.33		
R2	9.83	3.38–16.28			15.74	9.19–22.29			36.99	26.26–47.72		
**ACAT2 expression**												
Low	14.13	9.32–18.94	4.06	0.044	19.79	15.23–24.35	4.24	0.039	52.40	41.47–63.33	4.04	0.044
High	8.57	6.13–11.02			14.12	11.63–16.60			36.89	30.31–43.47		

**Note**: FIGO: International Federation of Gynecology and Obstetrics; CA125: carbohydrate antigen 125; HE4: human epididymal protein 4; ACAT2: Acetyl-CoA acetyltransferase 2; PFI: platinum-free interval; PFS: progression-free survival; OS: overall survival; CI: confidence interval

FIGO stage (*P* = 0.012), chemo-response (*P* < 0.001), ascites volume (*P* = 0.048), surgical satisfaction (*P* = 0.002) and ACAT2 expression (*P* = 0.044) were significantly related to PFI.

FIGO stage (*P* = 0.001), chemo-response (*P* < 0.001), surgical satisfaction (*P* = 0.005) and ACAT2 expression (*P* = 0.039) were significantly associated with PFS, while ascites volume had a significantly marginal effect on PFS (*P* = 0.074).

Pathological type (*P* = 0.004), chemo-response (*P* < 0.001), HE4 (*P* = 0.022), ascites volume (*P* = 0.013), surgical satisfaction (*P* = 0.025) and ACAT2 expression (*P* = 0.044) had significantly negative effects on OS, while FIGO stage produced a slight influence on OS (*P* = 0.079).

### Multivariate analysis of prognosis for PFI, PFS and OS

**Fig. 2:**
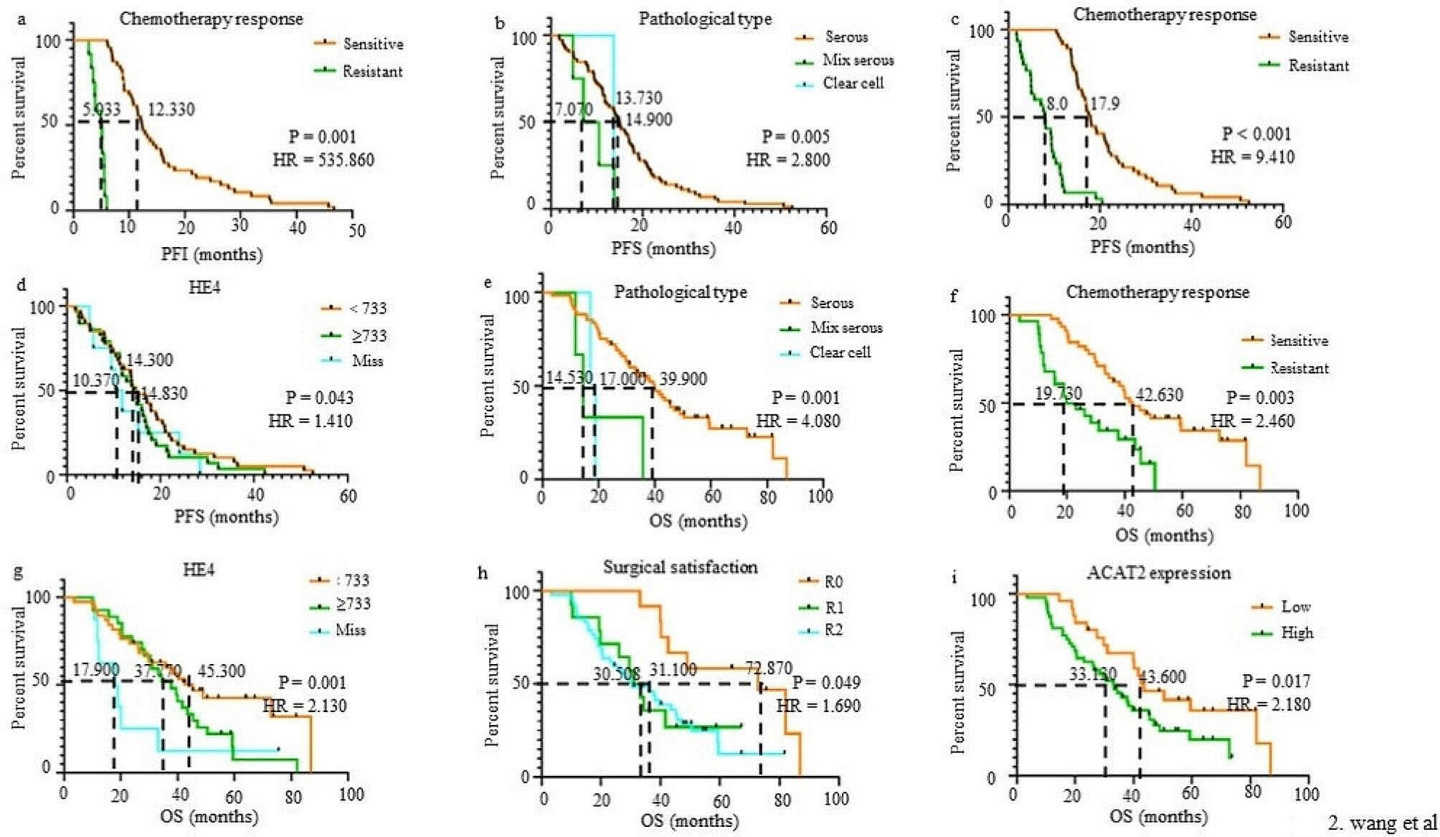
Kaplan-Meier survival curves for platinum-free interval (PFI), progression-free survival (PFS) and overall survival (OS) stratified by clinicopathological parameters in EOC; (**a**) was stratified by chemo-response for PFI; (**b-d**) were stratified by pathological type, chemo-response and HE4 for PFS; (**e-i**) were stratified by pathological type, chemo-response, HE4, surgical satisfaction and ACAT2 expression for OS

The independent prognostic factors for survival outcomes were analyzed by Cox-regression and displayed in Table 3. The survival plots were presented in Fig. 2. Results showed that chemo-response (hazard ratio (HR) = 535.86, *P* = 0.001) was an independent prognostic factor of PFI (Table 3 and Fig. 2a). Pathological type (HR = 2.80, *P* = 0.005), chemo-response (HR = 9.41, *P* < 0.001) and HE4 (HR = 1.41, *P* = 0.043) had independent effects on PFS (Table 3 and Fig. 2b-d**)**. Pathological type (HR = 4.08, P = 0.001), chemo-response (HR = 2.46, *P* = 0.003), HE4 (HR = 2.13, *P* = 0.001), surgical satisfaction (HR = 1.69, *P* = 0.049) and ACAT2 expression (HR = 2.18, *P* = 0.017) also affected OS independently (Table 3 and Fig. 2e-i).

**Table 3:** Prognostic factors for platinum-free interval (PFI), progression-free survival (PFS) and overall survival (OS) selected by cox regression

	PFI			PFS			OS		
	HR	95% CI	*P*	HR	95% CI	*P*	HR	95% CI	*P*
Pathological type	/	/	/	2.80	1.37–5.71	0.005	4.08	1.84–9.02	0.001
Chemo-response	535.86	15.20-18896.71	0.001	9.41	5.23–16.94	＜0.001	2.46	1.35–4.46	0.003
HE4	/	/	/	1.41	1.01–1.97	0.043	2.13	1.37–3.33	0.001
Surgical satisfaction	/	/	/	/	/	/	1.69	1.00-2.86	0.049
ACAT2 expression	/	/	/	/	/	/	2.18	1.15–4.11	0.017

*Note*: HE4: human epididymal protein 4; ACAT2: Acetyl-CoA acetyltransferase 2; PFI: platinum-free interval; PFS: progression-free survival; OS: overall survival; HR: hazard ratio; CI: confidence interval

## Discussion

Platinum-resistance is a major factor leading to shorter survival of OC [3]. Many researches [3–5] have concentrated on exploring the mechanism of platinum-resistance in OC, but rare remarkable progression has been made. ACAT2 is a new biomarker related to cancer [26–29]. Scarce studies of ACAT2 have been reported in OC. In this study, we verified high-expressed ACAT2 was significantly associated with chemo-resistance, advanced FIGO stage and decreased PFI, PFS and OS (all *P* < 0.05). Until now, no research has focused on this point.

Recent years, studies have shown that ACAT2 is aberrantly expressed in tumor, such as hepatocarcinoma [26], clear cell renal cell carcinoma [27], colorectal cancer [28] and breast cancer [29]. Weng’s study proved that high-expressed ACAT2 was related to advanced clinical stage and poor OS [32]. While in Zhao’s research [33], high-expressed ACAT2 was related to early tumor stage and longer survival, which was contradicted with ours. The heterogeneity of tumor may cause the above differences. Rare studies have reported the relationship between ACAT2 expression and chemo-resistance or ovarian cancer. However, Chemo-response was an independent prognosis factor of PFI, PFS and OS, which was consistent with the results of Hsiao-Yun Lu’s [14], Danielle Ikoma’s [34] and Jesus Gonzalez Bosquet’s [35]. Multivariate analysis showed that ACAT2 expression was an independent risk factor for OS of EOC, but not for PFI and PFS. A study about clear cell renal cell carcinoma suggested that ACAT2 expression was not an independent prognostic factor of survival [33]. This conclusion still requires more large-scale studies to confirm.

However, it is essential to explore the mechanism of chemo-resistance induced by ACAT2 in OC. At present, studies about ACAT2 expression mainly concentrate on the proliferation, migration and invasion of tumors [29, 32], lipid metabolism [25, 26] and radiation resistance [36]. A study has reported ACAT2 is a target for treatment of coronary heart disease related to hypercholesterolemia [37]. Souchek’s study proved that high-expressed ACAT2 was related to pancreatic cancer radiation resistance, which could be used as a novel target for radiotherapy sensitization [36]. No researchers have focused on the mechanism of chemo-resistance caused by aberrant ACAT2 expression. A study about Parkinson’s disease revealed that member 9 of the 70 kDa Heat Shock Protein (HSPA9) might be a potential interactant of ACAT2 by tandem affinity purification/mass spectra [38]. Related researches demonstrated HSPA9 was overexpressed in platinum-resistant OC [39] and breast cancer [40], and participated in resistance through P53 signaling pathway. Thus, we predicted the direct interaction between ACAT2 and HSPA9 existed, and further confirmed our hypothesis through online bioinformatics websites (https://cn.string-db.org/) (see supplement Fig. 1.). A published study revealed the upregulation of ACAT2 in chemo-resistant OC tissue mainly depended on an epigenetic approach of DNA hypomethylation [1]. Thus, we speculate that methylated ACAT2 participates in ovarian cancer chemo-resistance by acting on the HSPA9/P53 signaling pathway, which needs to be further explored.

Our study had some limitations. First, the small sample size of non-serous carcinoma and FIGO I-II in our study biased the research results; Second, rare studies between ACAT2 expression and chemo-response further confirmed our conclusions; Third, we failed to thoroughly explore the mechanism of ACAT2 overexpression leading to chemo-resistance, which would be our next research purpose; Last, more large-scale studies are needed to validate the relationship between ACAT2 expression and chemo-response in the future.

## Conclusion

The expression of ACAT2 in A2780/DDP and OVCAR8/DDP was higher than the corresponding A2780 and OVCAR8. High-expressed ACAT2 was associated with advanced FIGO stage, chemo-resistance, and decreased PFI, PFS and OS. Multivariate analysis demonstrated that ACAT2 expression was an independent prognosis factor of OS in EOC.

### Electronic supplementary material

Below is the link to the electronic supplementary material.

Supplementary Material 1

## Data Availability

The data in this article are available in the text and supplementary.

## Abbreviations

ACAT2: Acetyl-CoA acetyltransferase 2
EOC: epithelial ovarian cancer
PFI: platinum-free interval
PFS: progression-free survival
OS: overall survival
OC: ovarian cancer
IC50: half inhibitory concentration
CCK8: cell counting kit-8
FIGO: International Federation of Gynecology and Obstetrics
CA125: carbohydrate antigen 125
HE4: human epididymal protein 4
HR: hazard ratio
CI: confidence interval
