# Supplementary figures and images for: High-expressed ACAT2 predicted the poor prognosis of platinum-resistant epithelial ovarian cancer
Source: Diagn Pathol. 2024 Jan 4;19:7. doi: 10.1186/s13000-023-01435-4 (PMC10768435; doi:10.1186/s13000-023-01435-4)

**Supplementary**

**Fig. 1**. **The interaction diagram of ACAT2, HSPA9 and P53 from the STRING website.**


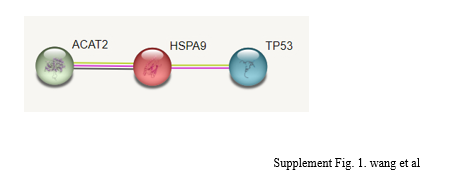

Supplement: Supplementary file 1 — Supplementary Material 1 [file 13000_2023_1435_MOESM1_ESM.docx]
